# Atorvastatin helps preserve pancreatic β cell function in obese C57BL/6 J mice and the effect is related to increased pancreas proliferation and amelioration of endoplasmic-reticulum stress

**DOI:** 10.1186/1476-511X-13-98

**Published:** 2014-06-21

**Authors:** Zhi-yu Chen, Shuai-nan Liu, Cai-na Li, Su-juan Sun, Quan Liu, Lei Lei, Li-hui Gao, Zhu-fang Shen

**Affiliations:** 1 Department of pharmacology, State Key Laboratory of Bioactive Substance and Function of Natural Medicines, Institute of Materia Medica, Chinese Academy of Medical Sciences and Peking Union Medical College, No.1 Xiannongtan Street, 100050 Beijing, P. R. China

**Keywords:** Atorvastatin, Diabetes, Pancreatic β cell function, Proliferation, ER stress, Apoptosis

## Abstract

**Background:**

3-Hydroxy-3-methyl-glutaryl CoA (HMG-CoA) reductase inhibitors or statins are competitive inhibitors of the rate-limiting enzyme in cholesterol biosynthesis. Currently, statins are used as first-line therapy in the treatment of diabetic dyslipidemia. However, effects of statins on β cell function remains unclear. This study aims to examine effects of atorvastatin treatment on pancreatic β cell function in obese C57BL/6 J mice and the possible mechanisms.

**Methods:**

Diet-induced obesity (DIO) C57BL/6 J mice were treated with atorvastatin (30 mg/kg/day) for 58 days. β cell function was assessed by hyperglycemic clamp and the area of insulin-positive β cells was examined by immunofluorescence. Gene expression was assessed by RT-PCR, and endoplasmic reticulum (ER) stress related proteins were examined by Western blot. Additionally, cell viability and apoptosis of the cholesterol-loaded NIT-1 cells were investigated after atorvastatin treatment.

**Results:**

Hyperglycemic clamp study revealed that glucose infusion rate (GIR) and insulin stimulation ratio in atorvastatin-treated DIO mice were markedly higher than control mice (P < 0.05, P < 0.01 vs. con), indicating preserved β-cell sensitivity to glucose. Lipid profiles of plasma triglyceride (TG), pancreas TG and plasma cholesterol (CHO) were improved. Pancreas weight and weight index were improved significantly after atorvastatin treatment (P < 0.05 vs. con). Immunofluorescence results showed that atorvastatin-treated mice had significantly larger insulin-positive β cell area (P < 0.05 vs. con). Furthermore, RT-PCR and western blot showed that the mRNA and protein expression of pancreatic and duodenal homeobox 1 (Pdx1) in the pancreas were upregulated (P < 0.001, P < 0.01 vs. con). Moreover, the expression level of ER stress markers of activating transcription factor 4 (ATF4), CCAAT-enhancer-binding protein homologous protein (CHOP) and phosphorylated eukaryotic initiation factor 2α (eIF2α) were downregulated in the pancreas of atorvastatin-treated mice (P < 0.001, P < 0.01, P < 0.01 vs. con). Besides, atorvastatin protected the pancreatic β cell line of NIT-1 from cholesterol-induced apoptosis. Western blot showed increased expression of anti-apoptotic protein of B-cell lymphoma 2 (Bcl-2).

**Conclusion:**

Pancreatic β cell function of obese C57BL/6 J mice was preserved after atorvastatin treatment, and this improvement may be attributed to enhanced pancreas proliferation and amelioration of pancreatic ER stress.

## Background

Statins are potent inhibitors of cholesterol biosynthesis. This class of agents have been used as lipid-altering agents, and exhibit beneficial effects on reducing cardiovascular risks [1-3]. Meanwhile, as the risks of cardiovascular disease are elevated in type 2 diabetes mellitus [4], statin therapy is indicated in metabolic syndrome and diabetic patients with cardiovascular risks [5,6].

Thus, whether statin therapy affects development of diabetes and which aspect of diabetes it will affect is intriguing. Pathological factors such as insulin resistance and β-cell failure have to be taken into account. On the one hand, recent studies showed that beneficial effect of atorvastatin on insulin resistance were due to decrease of inflammation [7]. On the other hand, functional β-cell mass was expanded with atorvastatin in the neonatal rodent [8]. However, whether the β-cell function will be affected is a concern. Clinical study of DIATOR trial showed that atorvastatin was effective in slowing the decline of beta cell function [9]. Besides, diabetes hazard was reduced by 30% in WOSCOPS trial [10]. Hence, we hypothesized that statin would positively affect β-cell function.

Many factors contribute to β-cell dysfunction, such as ER stress and mitochondrial dysfunction. It is reported that ER stress plays pivotal roles in both insulin resistance and β-cell failure. Markers of ER stress are elevated in the liver and adipose tissue in diet-induced forms of obesity and insulin action is interfered [11]. As ER serves as the protein folding factory for insulin [12], higher demand for insulin biosynthesis and secretion caused by long term over-nutrition will probably induce ER stress, and gradually leads to β-cell failure [13]. Evidences of the correlation between ER stress and diabetes also come from observations that humans and mice that have mutations in ER stress markers of double-stranded RNA-dependent protein kinase (PERK) and eIF2α are severely diabetic [14,15]. The islets of diabetic db/db mice show increased eIF2α phosphorylation and up-regulation of ATF4 and CHOP, indicating the presence of ER stress [16]. As atorvastatin showed beneficial effects on improving insulin sensitivity, we hypothesized the burden of ER to secret insulin was decreased and ER stress might be alleviated.

In our study, we used the insulin-resistant obese C57BL/6 J mice to assess the effects of atorvastatin on β cell function, β cell apoptosis and ER stress. It is shown that atorvastatin- treated mice had enhanced lipid profiles, β cell sensitivity to glucose, β cell proliferation and ameliorated ER stress state compared to the control mice. Atorvastatin also protected NIT-1 β cell line from apoptosis induced by cholesterol and increased anti-apoptosis protein of Bcl-2. Taken together, atorvastatin treatment benefits pancreatic β cell function through improved proliferation and attenuated ER stress.

## Results

### Atorvastatin improves β-cell sensitivity to glucose and lipid profiles of obese C57BL/6 J mice

**Figure 1:**
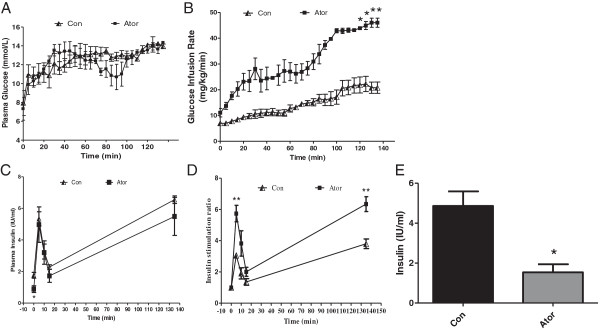
**Atorvastatin improves the** β**-cell sensitivity to glucose in obese C57 mice and decreases fasting insulin level.** Pancreatic beta cell function was evaluated by hyperglycemic clamp. After sampling (t = 0 min) for the assay of the basal blood glucose and insulin, animals received intravenously glucose bolus followed by a constant infusion of glucose to maintain plasma glucose level at 14 mmol/l. **(A)** Plasma glucose level, **(B)** Glucose infusion rates (GIR), **(C)** Plasma insulin level, **(D)** Insulin stimulation ratio, and **(E)** fasting insulin level. Results are means ± S.E.M. (n = 4-5). *P < 0.05, **P < 0.01 vs. control.

Using the hyperglycemic clamp, we examined the β-cell function. Relatively similar blood glucose concentrations of ~14 mmol/l of steady states were achieved by 135 min of glucose infusion in both groups (Figure 1A). In the atorvastatin-treated group, GIR which symbolizes the glucose metabolism was elevated approximately 2 fold (46.0 ± 1.8 mg/kg/min) compared with the control group (20.8 ± 2.2 mg/kg/min, P < 0.05 vs. controls) (Figure 1B). Fasting plasma insulin level of control mice was significantly higher than mice treated with atorvastatin (Figure 1C, P < 0.05), which indicated more severe insulin resistance state. The insulin secretion responses were biphasic, but the insulin stimulation rates at 5 minutes and steady state were much higher in atorvastatin group than control group (Figure 1D, P < 0.01). This implied the improved β-cell sensitivity to glucose after atorvastatin treatment. Besides, fasting insulin levels of the left C57BL/6 J mice in each group also showed that atorvastatin was beneficial for amelioration of insulin resistance (Figure 1E, P < 0.05 vs. con).

We further examined the lipid profiles of atorvastatin-treated mice. On day 58, plasma TG and pancreas TG were both diminished obviously (P < 0.05, P < 0.001 vs. con) in atorvastatin group (Table 1). Plasma CHO but not pancreas CHO decreased significantly (Table 1, P < 0.05). These results suggested that the lipid files were greatly improved in atorvastatin group.

**Table 1:** Lipid levels in plasma and pancreas of C57 mice at the end of atorvastatin treatment on day 58

**Group**	**Dose (mg/kg)**	**TG (mg/dl)**		**CHO (mg/dl)**	
		**Plasma**	**Pancreas**	**Plasma**	**Pancreas**
Nor	--	64.3 ± 5.6**	146.0 ± 23.4***	85.3 ± 5.9**	12.5 ± 1.5**
Con	--	140.0 ± 20.7	340.6 ± 7.9	270.2 ± 36.0	18.7 ± 1.8
Ator	30	84.0 ± 8.6*	212.3 ± 19.5***	170.5 ± 9.9*	18.2 ± 1.9

Values are mean ± S.E.M, n = 4-6. *P < 0.05, **P < 0.01, ***P < 0.001 vs. control.

### Atorvastatin increases pancreas weight and weight index, and helps improve insulin positive β-cell area

**Figure 2:**
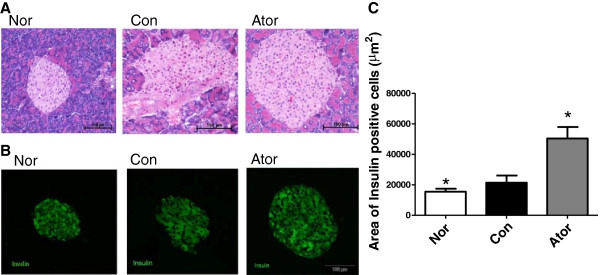
**Atorvastatin helps preserving** β**-cell area in obese C57 mice. (A)** H&E staining of pancreatic islets. **(B)** Fluorescent staining of insulin-expressing β-cells (FITC; 20×) in non-obese, obese mice with and without atorvastatin treatmen. **(C)** Quantification of area of insulin-expressing β-cells. Results are means ± S.E.M. (n = 3). *P < 0.05 vs. control.

We further examined the pancreas weight and calculated pancreas weight index. Pancreas weight was much heavier in atorvastatin group than control group (Table 2, P < 0.05), and the pancreas weight index was larger than that of control group (Table 2, P < 0.05). These results suggested atorvastatin might increase the pancreas proliferation. Thus, we observed the morphology of islet by H&E staining and immunofluorescence. The results showed that the area of insulin-positive granule of obese C57BL/6 J mice was significantly enlarged with irregular shape compared to the non-obese control mice, underlying the existence of compensatory hypertrophy of islets. Atorvastatin treatment could improve the shape of islets and increase the area of insulin positive granule, suggesting that atorvastatin might increase the insulin biosynthesis and content. (atorvastatin 50389.2 ± 7494.8 μm^2^ vs. control 21337.9 ± 3151.8 μm^2^, P < 0.5, Figure 2A-C).

**Table 2:** Effects of atorvastatin on pancreas/weight index of C57 mice at the end of the experiment

**Group**	**Dose (mg/kg)**	**Body weight (g)**	**Pancreas weight (g)**	**Pancreas weight index (×10-3)**
Nor	--	24.2 ± 0.5***	0.147 ± 0.007***	6.1 ± 0.4*
Con	--	47.5 ± 2.1	0.232 ± 0.016	4.8 ± 0.4
Ator	30	41.5 ± 2.1	0.279 ± 0.006*	6.8 ± 0.6*

Results are means ± S.E.M, n = 4-5. *P < 0.05, ***P < 0.001 vs. control.

### Atorvastatin up-regulates Pdx-1 and LXR-β gene expression and suppresses ER stress by downregulating the peIF2α-ATF4-CHOP pathway

**Figure 3:**
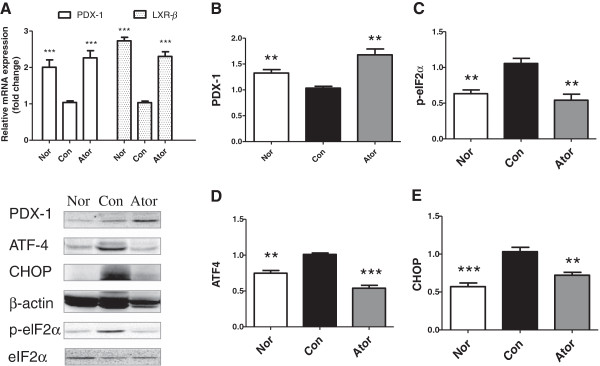
**Atorvastatin upregulates PDX-1 and LXR-β expression and downregulates the protein expressions of ER stress markers.** Total RNA was extracted from the pancreas of C57 mice and analyzed by quantitative real-time PCR. A comparative threshold cycle (CT) method was used for relative quantification of gene expression using beta-actin for normalization. Measurements were carried out in triplicate for each sample. **(A)** Relative mRNA levels of PDX-1 and LXR-β in pancreatic cells. Western blot analysis of pancreatic **(B)** PDX-1 **(C)** phosphorylated eIF2α **(D)** ATF4 **(E)** CHOP in C57 mice compared in three groups. Beta actin served as loading control. Data represented the mean of at least three independent experiments ± S.E.M. **p < 0.01, ***p < 0.001 vs. control.

To determine whether there is a specified mechanism of effects of atorvastatin, we examined the pancreas gene expression. Pdx-1, a critical molecule for pancreatic cell proliferation and Liver X receptor β (LXR-β), which plays a crucial role in the control of lipid metabolism were significantly up-regulated by atorvastatin (Figure 3A, P < 0.001). Western blot further confirmed that the protein level of Pdx-1was markedly higher in the pancreas of atorvastatin treated mice compared with the control mice (Figure 3B). These findings suggested that atorvastatin upregulated molecules critical for pancreatic cell proliferation and lipid metabolism.Furthermore, western blot revealed that eIF2α, which is downstream of PERK and is phosphorylated by PERK in the unfolded protein response (UPR), was less phosphorylated in the pancreas of normal and atorvastatin-treated mice compared with the control mice (P < 0.01, Figure 3C). We further examined the effect of atorvastatin on the expression of ATF4 and CHOP in pancreatic tissues. We found that, consistent with the result of reduced phosphorylation of eIF2α, the protein levels of ATF4 and CHOP were both down-regulated significantly in the atorvastatin-treated mice compared with the control mice (Figure 3D, E, P < 0.001, P < 0.01 vs. con). These findings implied the existence of ER stress state in pancreas of obese C57BL/6 J mice, and atorvastatin could attenuate the ER stress by modulating the peIF2α-ATF4-CHOP pathway.

### Atorvastatin increases survival of NIT-1 cells under the apoptosis induced by cholesterol loading

**Figure 4:**
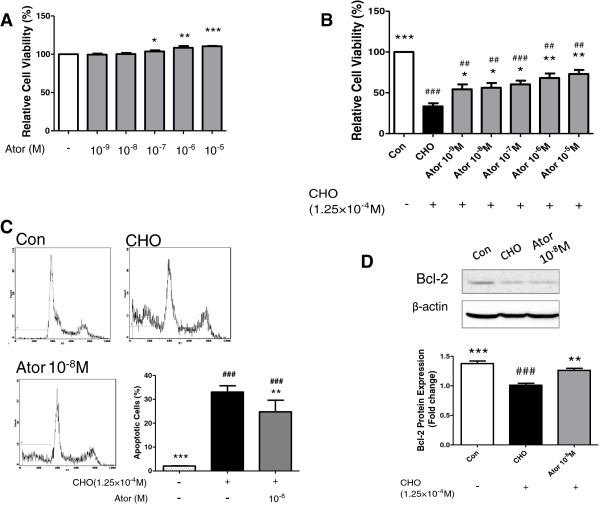
**Atorvastatin attenuates cholesterol-induced apoptosis of NIT-1 cells. (A)** Effects of atorvastatin alone on NIT-1 cell viability. (n = 3–5) **(B)** Reserved β-cell relative viability after addition of different concentrations (10^-9^ to 10^-5^ M) of atorvastatin under treatment with 0.125 mM cholesterol for 12 h. Values are means ± S.E.M. (n = 3). **(C)** NIT-1 cell apoptosis with or without treatment of cholesterol and in addition of 10^-8^ M atorvastatin incubation for 18 h. Results were detected by flow cytometry and quantification was made based on propidium iodide (PI) positive cells. (n = 3) **(D)** Protein expression of anti-apoptotic Bcl-2 in the pancreas. All the data were expressed as mean ± S.E.M. *P < 0.05, **P < 0.01, ***P < 0.001 vs. the CHO group; ^##^P < 0.01, ^###^P < 0.001 vs. control.

Although proliferative effect was shown after atorvastatin treatment, it remains unclear whether the expansion of β-cells was also due to decreased apoptosis. Thus, we investigated whether atorvastatin modulates the apoptotic response in pancreatic β cell line NIT-1. First of all, we examined whether atorvastatin affects the cell viability of NIT-1 cells. Results showed that no adverse effects were exerted on NIT-1 cells viability, and atorvastatin 10^-7^ M-10^-5^ M significantly increased NIT-1 cells viability (Figure 4A). This proliferative effect at high concentration is corresponding with increased β-cell area observed in C57 mice. Treatment with 0.125 mM cholesterol for 12 h reduced the viability of NIT-1 cells by 67% (Figure 4B, P < 0.001). Atorvastatin (10^-9^-10^-5^ M) improved NIT-1 cells viability treated with 0.125 mM cholesterol in a dose-dependent manner (P < 0.05 for 10^-9^-10^-7^ M, P < 0.01 for 10^-6^-10^-5^ M, Figure 4B). The viabilities of NIT-1 cells treated with 10^-9^ M and 10^-5^ M atorvastatin were 163% and 219% higher than NIT-1 cells treated with cholesterol alone. Flow cytometry analysis further revealed that cholesterol-treated NIT-1 cells had an apoptotic rate of 33 ± 2.1%, which was reduced to 24 ± 3.8% with 10^-8^ M atorvastatin treatment (P < 0.01, Figure 4C). Western blot additionally revealed that atorvastatin induced up-regulation of anti-apoptosis protein of Bcl-2 in NIT-1 cells, and this might contribute to the increased NIT-1 cells survival (P < 0.01, Figure 4D).

## Discussion

In this study, the effects of atorvastatin treatment on pancreatic β cell function in obese C57BL/6 J mice and its possible mechanism were investigated. After 58 days of treatment, GIR and insulin stimulation ratio of atorvastatin treated mice were improved compared with control mice. Furthermore, these mice had greater insulin positive β cell area. Besides, pancreas ER stress markers were down-regulated. In vitro results suggest a protective role of atorvastatin against cholesterol-induced apoptosis of NIT-1 cells. All the results indicate the beneficial effects of atorvastatin on β-cell function.

The hyperglycemic clamp has been demonstrated to be a reliable technique to evaluate β-cell sensitivity to glucose [17]. Because the glucose level is held constant, the glucose infusion rate is an index of glucose metabolism. Defect of initial phase of insulin secretion is the earliest detectable abnormality in diabetes mellitus. The insulin stimulation ratios of first and second phases were both enhanced after atorvastatin treatment, indicating a preserved β cell function.

Up to date, many researchers have showed the beneficial effects of atorvastatin on insulin resistance were due to amelioration of inflammation [7,18,19]. In hyperglycemic clamp test, the insulin at 0 min in atorvastatin group was significantly lower (p < 0.05 vs. con), and we confirmed the result by examining insulin level again when the animals were sacrificed. The decreased fasting insulin implied the insulin resistance was ameliorated and this is accordant to results reported above. It is possible that the decreased demand of insulin could relieve the work burden of ER to synthesize and secrete insulin. Hence, the ER stress state was alleviated based on lipid-independent effect.

We observed that 30 mg/kg atorvastatin improved the lipid profile of obese C57BL/6 J mice. Plasma TG and pancreatic TG were both decreased (P < 0.05, P < 0.001 vs. Con ). TG is reported to activate ER stress, and ER stress impairs insulin sensitivity by decreasing the tyrosine phosphorylation of insulin receptor substrate-1 (IRS-1) [20]. Thus, the lipid-lowering effect of atorvastatin may lead to amelioration of ER stress and insulin resistance and eventually preserved the β-cell function. Moreover, the LXR-β, which plays a central role in regulation of cholesterol metabolism was also up-regulated in pancreas.

On the other hand, atorvastatin may improve β cell function through accelerating the pancreas proliferation as shown by the immunofluorescence results. PDX-1 mRNA and protein level in pancreas were both increased significantly. PDX-1 is important for pancreatic development and β-cell function. Pdx1^+/-^ mice showed worsening glucose tolerance and insulin secretion and the islets were more susceptible to apoptosis [21].

Another possible reason of preserved β cell function could be the improvement of endothelial function. Islet endothelium plays an important role in providing oxygen and nutrients to endocrine cells, trans-endothelial rapid passage of secreted insulin into the circulation and blood glucose sensing and regulation [22,23]. Endothelial dysfunction has been shown in patients with type 1 and type 2 diabetes mellitus. Improved metabolic control in diabetic patients is associated with near restoration of endothelial function [24]. As statins were shown to increase the expression of eNOS and iNOS [25,26], and may increase the NO production leading to vascular relaxation. Endothelium function may therefore be improved. Atorvastatin was reported to improve the regeneration of β-cell mass due to increase of intro-islet endothelial cells [8].

As the strong association between the ER stress and diabetes [27,28], we specifically investigated whether atorvastatin exerted its effect on pancreatic β cells by modulating the ER stress. Once the ER stress is present, the UPR will be triggered to cope with stress conditions. There are three sensing proteins, inositol-requiring 1α (IRE1α), PERK and activating transcription factor 6 (ATF6) [29]. As for the PERK pathway, PERK phosphorylates eIF2α, and this will cause more efficiently translation of ATF4. Chop is the downstream protein of PERK–eIF2α–ATF4 pathway and mainly induce the apoptosis caused by the ER stress in the UPR [30]. Moreover, Chop^-/-^ mice had improved glycemic control and expanded beta cell mass [31]. In this study, we found that eIF2α–ATF4-Chop pathway was turned down after atorvastatin treatment. However, whether ATF6 and IRE1α pathways are involved needs to be investigated in further study.

The cholesterol induced apoptosis model of NIT-1 cells provides us a tool to investigate the effects of atorvastatin. Recently, cholesterol has been proved to induce the ER stress and apoptosis in macrophages [32]. As type 2 diabetes is accompanied with inflammation, we mimicked the ER stress state by loading cholesterol onto NIT-1 cells. We found that cholesterol suppressed the viability of NIT-1 cells, which was attenuated by atorvastatin in a dose-dependent manner. Flow cytometry test further demonstrated that atorvastatin ameliorated cholesterol-induced apoptosis of NIT-1 cells. CHOP was shown to down-regulate the anti-apoptotic protein of Bcl-2 [33], the preserved Bcl-2 expression in NIT-1 cells is accordant with the depression of CHOP expression in pancreas. Besides, atorvastatin alone did not negatively affect NIT-1 cell viability and increased viability at high concentration. This result could interpret the increased insulin positive β-cell area observed in C57 mice.

However, some clinical trials have revealed the deterioration of glucose metabolism of statins [6]. And FDA has expanded advice on statin risks of possibility of developing type 2 diabetes. The possibility that patients who have cardiovascular disease are already at high risk of developing diabetes can’t be expelled. Another explanation is that long term aggressive therapy with statins could induce the adverse effects.

In this study, aggressive dose of atorvastatin was used. The dose of 30 mg/kg/d of atorvastatin in mice is equivalent to 170 mg/d in a 70 Kg human calculated based on the body surface area (BSA) [34,35]. This is more than the highest dose of 80 mg/d which is recommended. For adults with diabetes, the American Diabetes Association recommends aggressive use of statin in the treatment of diabetic dyslipidemia [36]. In the REVERSAL trial, aggressive lipid lowering with atorvastatin (80 mg/d) showed beneficial effects on halting the atherosclerosis progression (-0.4%) compared with baseline, and the effect is superior to simvastatin 40 mg [37]. Meanwhile, antioxidant and anti-inflammation benefits of atorvastatin 80 mg were also observed in MIRACL and ASAP trails [38,39]. Besides, the aggressive (80 mg/d) and moderate (10 mg/d) lipid-lowering therapy with atorvastatin was compared in the DALI trail [40]. Consequently, Fasting TG was reduced by 35% with aggressive therapy and by 25% with moderate therapy. Thus, atorvastatin 80 mg/d is of better effects on lipid file alterations compared with 10 mg/d.

## Conclusion

In conclusion, treatment of insulin-resistant obese C57BL/6 J mice with atorvastatin exhibits a protective effect on pancreatic β cell function and this is related to increased pancreas proliferation and decreased ER stress. Our finding is expected to provide evidence for a better and appropriate clinical use of atorvastatin.

## Methods

### Cells

The pancreatic β cell line NIT-1 was purchased from ATCC (Manassas, VA) and cultured in DMEM/F12 containing 10% (v/v) fetal bovine serum (FBS) and 1% (v/v) antibiotics (100 U/mL penicillin and 0.1 mg/ml streptomycin) at 37°C in a humidified atmosphere containing 5% CO_2_[41].

### Animals and hyperglycemic clamp studies

All animals were handled in accordance with The Standards for Laboratory Animals (GB14925-2001) and The Guideline on the Humane Treatment of Laboratory Animals (MOST 2006a) established by the People’s Republic of China. The two guidelines were conducted in adherence to the regulations of Institutional Animal Care and Use Committee (IACUC) and all animal protocols were approved by IACUC.

Six-week-old male C57BL/6 J mice (Institute of Laboratory Animal Science, CAMS and PUMC, Beijing, China) were housed in a controlled environment with a 12 h light/dark cycle (lights on from 6:00 AM to 6:00 PM) at constant temperature (22–25°C) with ad libitum access to food and water. Mice were fed with high-fat diets for 20 weeks and were then randomly assigned to receive atorvastatin at 30 mg/kg/day or vehicle for 58 days by gavage with 10 mice per group. High fat diet is kept during the 58 days. Hyperglycemic studies were conducted as described previously [42]. On day 52, after a 10-h fast, mice (4 to 5 from each group) were anesthetized with pentobarbital sodium (50 mg/kg body weight, intraperitoneally) and placed on a heating pad at 37°C. The right jugular vein was catheterized (Micro-renathane, 0.025 × 0.012″) and after a 30 min rest, the mice received a bolus injection of D-glucose at 0.25 g/kg body weight followed by continuous infusion of glucose (50%) at 10–20 μL/min. Blood samples were collected initially (0 min) and then every 5 or 10 min from the tail throughout the test for determination of glucose concentration using a glucose meter (ACCU-CHEK Active; Roche, Mannheim, Germany). The infusion rate of glucose was adjusted according to the results of plasma glucose concentration to achieve a steady state with hyperglycemia nearly 14 ± 0.5 mmol/l. At the end of the clamp, glucose infusion rate (GIR) was calculated. On day 58, the remaining mice were sacrificed for additional assays. Body weight and pancreas were weighed and the pancreas weight index was calculated using the formula below. Pancreas weight index = pancreas weight (g)/body weight (g)

### Histological studies

Pancreatic tissue specimens were fixed in 10% formalin overnight and then paraffin-embedded and sectioned at a thickness of 7 μm. The tissue sections were deparaffinized and rehydrated sequentially in xylene, xylene/ethanol, and gradient ethanol, and then placed in distilled water for 10 min. Pancreatic tissue sections were then stained with hematoxylin and eosin (H&E) using standard protocols.

### Immunofluorescence

Paraffin-embedded pancreatic tissue sections were dewaxed using xylene, rehydrated through gradient alcohol [43]. The sections were washed and incubated with mouse anti-insulin antibody (Santa Cruz Biotechnology, Santa Cruz, CA) and then FITC-conjugated goat anti-mouse IgG (Zhongshan Jinqiao Co., Beijing, China). Images were obtained using a Leica TCS SP2 laser scanning confocal microscope (Nikon) and analyzed using the Image pro plus 5.1 image analysis software (Media Cybernetics, Silver Spring, MD, USA). Three animals were included in each group. At least three sections from each animal were analyzed. For each section, the area of insulin positive β-cell in each islet was determined and the average area was calculated.

### Quantitative Real time PCR

Total cellular RNA was extracted from the pancreatic tissue of C57BL/6 J mice using Trizol reagent (Invitrogen, Carlsbad, CA). Reverse transcription reactions for the preparation of first strand cDNA was performed using VigoScript First Strand cDNA Synthesis Kit (Vigorous Biotech- nology Beijing Co., Ltd.). RNase-Free DNase (Promega, Madison, WI) was used to degrade DNA prior to qPCR detection. Quantitative real time PCR (qPCR) was performed on an ABI 7000 Real Time PCR system (Applied Biosystems, Foster City, CA) using the SYBR Premix Ex Taq kit (TakaRa, Japan). All samples were analyzed in triplicate and normalized with β-actin used as an internal control. The primer sequences were as follows: β-actin, 5′- AGAAGATCTGGCACCACACC 3′(sense) and 5′-TACGACCAGAGGCATACAGG-3′ (antisense); Pdx-1, 5′-CCCGAATGGAACCGAGCCT-3′ (sense) and 5′-CCCGAGGTCACCGCACAAT-3′ (antisense); LXR-β, 5′-AAGGACTTCACCTACAGCAAGGA-3′ (sense) and 5′–GAACTCGAAGATGGGATTGATGA-3′ (antisense).

### Western blot

Pancreatic tissue homogenates were prepared in lysis buffer (50 mM Tris–HCl, 2% SDS, and 10% glycerol) supplemented with a protease inhibitor cocktail (Applygen Inc. Beijing, China) as previously described [44]. Additionally, cellular lysates of NIT-1 cells were prepared as previously depicted [45]. Proteins were resolved by SDS-PAGE and immunoblotting assays were performed as earlier mentioned [21]. The following antibodies were used (1:1000 dilution unless otherwise indicated): total eIF2α (sc-11386, 1:500), ATF4 (sc-200), PDX-1 (sc-25403), and CHOP (sc-575), Bcl-2 (sc-7382) (all from Santa Cruz Biotechnology), phospho-EIF2α (Ser51, 9721) (Cell Signaling Technology, Danvers, MA) and β-actin (Abmart, 1:2000). Protein bands were visualized by chemiluminescence (ChemiScope2850, CLiNX Science Instruments) and density was analyzed using the Gel-Pro-Analyzer 3.1 software.

### Biochemical analysis

Plasma triglycerides (TG), pancreas TG, plasma cholesterol (CHO) and pancreas CHO were determined by enzymatic colorimetric methods using commercial kits (BioSino Inc., China). Plasma insulin was measured by ELISA (Alpco. Inc., USA).

### Cell viability and apoptosis assays

NIT-1 cells were seeded into 96-well plate at 2.3 × 10^4^ cells/well, and cultured in DMEM/F12 medium supplemented with 10% FBS. When cells reached 80% confluence, they were incubated with 0.125 mM water-soluble cholesterol (Sigma-Aldrich, St. Louis, MO) for 12 h in the absence or presence of atorvastatin at concentration from 10^-9^ to 10^-5^ M. Thereafter, NIT-1 cells were tested for viability by using CCK-8 kit (Dojindo Laboratories, Kumamoto, Japan). The study was conducted for 3 times with 5 wells in each group. For apoptosis assays, NIT-1 cells were seeded into 6-well plate at density of 4 × 10^5^ cells/well. When the cells were 80% confluent, they were incubated with 0.125 mM water-soluble cholesterol for 18 h with or without 10^-8^ M atorvastatin [46]. Then, the cells were digested and fixed with 70% ethanol and incubated with 50 μg/mL propidium iodide and 1 μg/mL DNase-free RNase. Stained cells were analyzed on a flow cytometer (Beckman-coulter, Brea, CA), and the number of late-stage apoptotic cells was analyzed by System II software.

### Statistical analysis

Data were expressed as the mean ± standard error of the mean (SEM). The data obtained in the present study were analyzed using an ANOVA. A p value < 0.05 was considered to be statistically significant.

## Abbreviations

DIO: Diet-induced obesity; ER: Endoplasmic reticulum; UPR: Unfolded protein response; PDX-1: Pancreatic and duodenal homeobox 1; LXR-β: Liver X receptor β; eIF2α: Eukaryotic initiation factor 2α; ATF4: Activating transcription factor 4; CHOP: CCAAT-enhancer-binding protein homologous protein; IRE1α: Inositol-requiring 1α; PERK: Double-stranded RNA-dependent protein kinase; ATF6: Activating transcription factor 6; CHO: Total cholesterol; TG: Triglyceride; GIR: Glucose infusion rate; Bcl-2: B-cell lymphoma 2.

## Competing interests

The authors declare that they have no competing interests.

## Authors’ contributions

ZYC carried out studies including Real-time PCR, Western Blot and immunofluorescence assays and all the data analysis. ZYC and SNL performed the clamp test. CNL, SJS and QL participated in the animal in vivo experimental test and biochemical analysis. LL participated in immunofluorescence assays and data analysis. LHG participated in the sequence alignment. ZYC and ZFS wrote the paper. ZFS designed the study and in coordination with all others drafted the manuscript. CNL participated in revising the manuscript. All authors read and approved the final manuscript.
